# Evaluating *BRCA* mutation risk predictive models in a Chinese cohort in Taiwan

**DOI:** 10.1038/s41598-019-46707-6

**Published:** 2019-07-15

**Authors:** Fei-Hung Hung, Yong Alison Wang, Jhih-Wei Jian, Hung-Pin Peng, Ling-Ling Hsieh, Chen-Fang Hung, Max M. Yang, An-Suei Yang

**Affiliations:** 1 Koo Foundation Sun-Yat Sen Cancer Center, Taipei, Taiwan; 2 Genomics Research Center, Academia Sinica, Taipei, Taiwan; 3 National Yang Ming University School of Medicine, Taipei, Taiwan; 4 University of Cincinnati College of Medicine, Cincinnati, Ohio, USA

**Keywords:** Breast cancer, Cancer models, Cancer genetics

## Abstract

Accurate estimation of carrier probabilities of cancer susceptibility gene mutations is an important part of pre-test genetic counselling. Many predictive models are available but their applicability in the Asian population is uncertain. We evaluated the performance of five *BRCA* mutation risk predictive models in a Chinese cohort of 647 women, who underwent germline DNA sequencing of a cancer susceptibility gene panel. Using areas under the curve (AUCs) on receiver operating characteristics (ROC) curves as performance measures, the models did comparably well as in western cohorts (BOADICEA 0.75, BRCAPRO 0.73, Penn II 0.69, Myriad 0.68). For unaffected women with family history of breast or ovarian cancer (n = 144), BOADICEA, BRCAPRO, and Tyrer-Cuzick models had excellent performance (AUC 0.93, 0.92, and 0.92, respectively). For women with both personal and family history of breast or ovarian cancer (n = 241), all models performed fairly well (BOADICEA 0.79, BRCAPRO 0.79, Penn II 0.75, Myriad 0.70). For women with personal history of breast or ovarian cancer but no family history (n = 262), most models did poorly. Between the two well-performed models, BOADICEA underestimated mutation risks while BRCAPRO overestimated mutation risks (expected/observed ratio 0.67 and 2.34, respectively). Among 424 women with personal history of breast cancer and available tumor ER/PR/HER2 data, the predictive models performed better for women with triple negative breast cancer (AUC 0.74 to 0.80) than for women with luminal or HER2 overexpressed breast cancer (AUC 0.63 to 0.69). However, incorporating ER/PR/HER2 status into the BOADICEA model calculation did not improve its predictive accuracy.

## Introduction

*BRCA* mutation carriers face 45% to 85% risk of developing breast cancer, and 10% to 46% risk of developing ovarian cancer by age 70^1–3^. With recent advances in gene sequencing technology, detecting germline mutations in cancer susceptibility genes is becoming more widely accessible to the public. Effective risk reducing surgeries, medications, and/or intense cancer screening strategies are available to *BRCA* mutation carriers to manage risks of breast and ovarian cancer at an early or even pre-emptive stage. These advances introduce the possibility of substantial benefits from broad genetic screens for individuals at risk for *BRCA* mutation^4^.

However, before genetic testing, accurate estimation of the probability of carrying a germline mutation in cancer susceptibility genes is crucial. Genetic testing in a low risk individual could lead to potential harm, dissatisfaction, and misallocation of resources^5,6^. In fact, many professional societies recommend at-risk individuals to receive genetic counselling before undergoing genetic testing^5,7–9^, where the probability of carrying a germline mutation must be determined to ascertain the risks and benefits of genetic testing.

A variety of risk predictive models have been developed to determine the probability of carrying a *BRCA* mutation utilizing personal and family cancer history and mathematical models^10–16^. A threshold risk rate could be set at the discretion of the clinician or genetic counsellor to determine whether the patient should proceed with genetic testing. These predictive models have been tested and utilized extensively in European and American geographies^17–20^. However, only a few studies have tested their performance in Asian populations, and those that have done so exhibited mixed results in discerning risk for *BRCA* mutations compared to their western counterparts. Several studies reported poor model performance in Asian breast cancer cohorts, including evaluation of Manchester Scoring System^14^ and BOADICEA^10^ in a Malaysian cohort of 187 patients with *BRCA* mutation rate of 14.4%^21^, and evaluation of BRCAPRO^11^ and Myriad^12^ in a Korean cohort of 236 patients with *BRCA* mutation rate of 19.5%^22^. In contrast, an updated Manchester Scoring System that included adjustment for breast cancer receptor status and high grade serous type ovarian cancer was shown to be equally effective in *BRCA* mutation prediction in the Singapore cohort as in the Manchester population^23^. In a cohort of 212 Chinese familial breast cancer patients with *BRCA* mutation rate of 15.6%, BRCAPRO, Penn II^16^, and Myriad models showed comparable accuracy to western cohorts^24^. In a study of Hong Kong Chinese cohort consisted of 310 female and male breast or ovarian cancer patients with *BRCA* mutation rate of 13.9%^25^, BOADICEA appeared to be the most accurate in combined *BRCA1/*2 mutation prediction among the five tested models, while BRCAPRO better predicted mutations of *BRCA1* alone. These two models actually performed slightly better in the Chinese cohort than in several western and Asian cohorts previously reported. BRCAPRO and Myriad models were also tested in a Korean ovarian cancer cohort of 232 with 24.6% *BRCA* mutation prevalence^26^, in which both models had acceptable performance. The aforementioned studies tested the models only in affected individuals with breast or ovarian cancer, and the cohorts were relatively small. More studies in Asian cohorts are clearly needed to validate and discern how best to use these models.

In the present study, we aimed to evaluate the mutation predictive accuracies of BOADICEA, BRCAPRO, Myriad, Penn II, and Tyrer-Cuzick models in those at risk for hereditary breast and ovarian cancer syndrome in a Chinese cohort in Taiwan. We explored model performances in various subgroups to determine how best to use the models in pre-test genetic counselling.

## Results

A total of 647 female participants from 488 families were included in the study. The cohort was divided into three subgroups based on the presence or absence of personal history and family history (FH) of breast cancer (BC) or ovarian cancer (OC). The personal characteristics, cancer characteristics, and mutation frequencies for the entire cohort and for the subgroups are shown in Table 1. The mean age at study enrolment was 50.2, ranging from 16 to 96. Among them, 503 (77.7%) had a personal history of BC or OC, and 385 (59.5%) had a family history of BC or OC. The subgroup with personal history but no family history of BC or OC (BC/OC(+)FH(−)) were younger (mean age 47.8) and included more early onset cancer and triple-negative breast cancer. In the entire cohort, 48 individuals were found to be carrying a *BRCA* mutation (12 *BRCA1*, 36 *BRCA2*), making the carrier rate of 7.4%. The subgroup with both personal and family history of BC or OC (BC/OC(+)FH(+)) had the highest *BRCA* mutation carrier rate of 10.4%.

**Table 1:** Participant characteristics.

	All Participants N = 647	BC/OC(+) FH(+) n = 241	BC/OC(+) FH(−) n = 262	BC/OC(−) FH(+) n = 144
Age, mean (range)	50.2 (16–96)	53.0 (28–82)	47.8 (18–89)	49.8 (16–96)
Personal history of BC/OC	503 (77.7%)	241 (100%)	262 (100%)	0 (0%)
Family history of BC/OC	385 (59.5%)	241 (100%)	0 (0%)	144 (100%)
**Mutations detected:**				
*BRCA1* mutation	12 (1.9%)	8 (3.3%)	2 (0.8%)	2 (1.4%)
*BRCA2* mutation	36 (5.6%)	17 (7.1%)	15 (5.7%)	4 (2.8%)
Other HR gene mutation	31 (4.8%)	14 (5.8%)	10 (3.8%)	7 (4.9%)
**In those with BC/OC:**	**n = 503**	**n = 241**	**n = 262**	**n = 0**
Age of onset ≤40 for BC/OC	265 (52.7%)	90 (37.3%)	175 (67.8%)	N/A
Triple-negative breast cancer	136 (27.0%)	50 (20.7%)	86 (32.8%)	N/A
Bilateral breast cancer	54 (10.7%)	33 (13.7%)	21 (8.0%)	N/A
Ovarian cancer	10 (2.0%)	5 (2.1%)	5 (1.9%)	N/A

All values are listed as number (% of the subgroup in each column), unless otherwise specified.

BC: breast cancer; OC: ovarian cancer; BC/OC(+): personal history of breast/ovarian cancer; FH(+): family history of breast/ovarian cancer; HR gene: homologous recombination pathway genes tested in this study other than *BRCA*: *ATM*, *BARD1*, *BRIP1*, *PALB2*, *RAD50*, *RAD51C* and *RAD51D* (detailed numbers are provided in the Supplementary Table 1S); N/A: not applicable; FH: family history, defined as two or more first-, second- or third-degree relatives on the same lineage of the family with BC or OC.

### Model performance by genes

**Figure 1:**
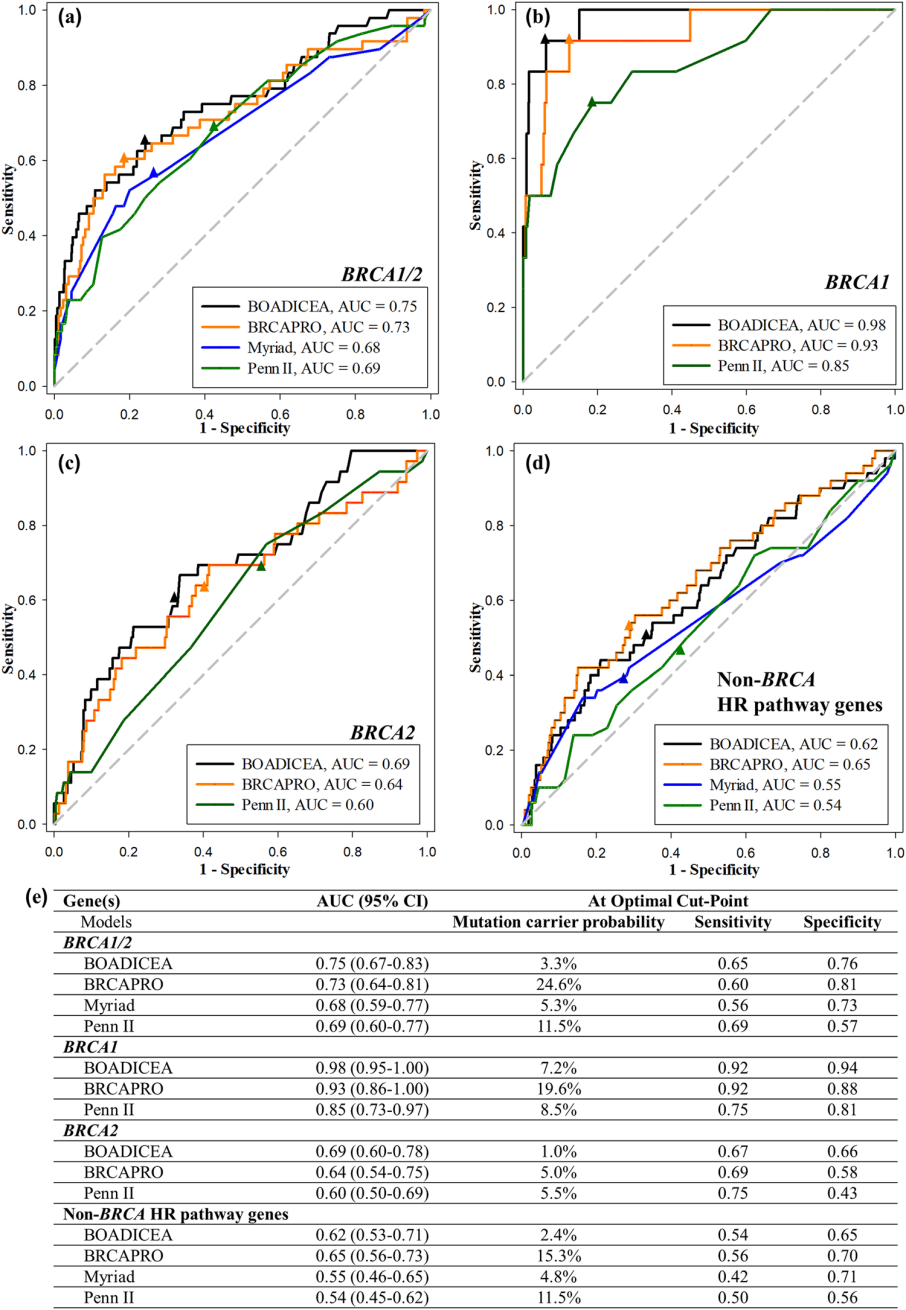
Performance of four *BRCA* mutation risk predictive models using ROC curves; the respective AUC for each model is shown at the right lower corner of the curves and in panel (**e**); the optimal cut-points (closest point to the left upper corner) are shown as triangles on each curve. (**a**) *BRCA1/2*: probability of *BRCA1* or *BRCA2* mutation prediction using the BOADICEA, BRCAPRO, Myriad, and Penn II models; (**b**) *BRCA1* only: probability of *BRCA1* mutation prediction using the BOADICEA, BRCAPRO, and Penn II models; (**c**) *BRCA2* only: probability of *BRCA2* mutation prediction using the BOADICEA, BRCAPRO, and Penn II models; (**d**) Non-*BRCA* HR pathway genes: probability of HR pathway gene other than *BRCA1/2* (*ATM*, *BARD1*, *BRIP1*, *PALB2*, *RAD50*, *RAD51C*, *RAD51D*) mutation prediction using the BOADICEA, BRCAPRO, Myriad, and Penn II models; (**e**) AUC with 95% confidence interval (CI) for each model and each gene set, as well as mutation carrier probability, sensitivity, and specificity at the optimal cut-point for each curve are listed.

Figure 1 shows the performance of four mutation predictive models on ROC curves. The AUCs for having either a *BRCA1* or a *BRCA2* mutation were: 0.75 (95% CI, 0.67–0.83) for BOADICEA, 0.73 (95% CI, 0.64–0.81) for BRCAPRO, 0.68 (95% CI, 0.59–0.77) for Myriad, and 0.69 (95% CI, 0.60–0.77) for Penn II (Fig. 1a). At the optimal cut-points, defined by the closest points to the left upper corner, the values of the mutation carrier probabilities varied widely, with BOADICEA having the lowest cut-off value of 3.3%, BRCAPRO having the highest cut-off value of 24.6%, and Myriad and Penn II having the middle values of 5.3% and 11.5%, respectively. The sensitivities at the optimal cut-points were between 0.56 and 0.69, while the specificities were between 0.57 and 0.81 (Fig. 1e).

Three of the models also predict the *BRCA1* and *BRCA2* mutation carrier probabilities separately. The models performed very well for *BRCA1* and worse for *BRCA2*. Figure 1b shows the AUCs for *BRCA1*-only predictions: 0.98 (95% CI, 0.95–1.00) for BOADICEA, 0.93 (95% CI, 0.86–1.00) for BRCAPRO, and 0.85 (95% CI, 0.73–0.97) for Penn II. The optimal cut-point values for *BRCA1* mutation carrier probability were: 7.2% for BOADICEA, 19.6% for BRCAPRO, and 8.5% for Penn II. Figure 1c shows the ROC curves for *BRCA2*-only prediction, and the AUCs were: 0.69 (95% CI, 0.60–0.78) for BOADICEA, 0.64 (95% CI, 0.54–0.75) for BRCAPRO, and 0.60 (95% CI, 0.50–0.69) for Penn II. The optimal cut-point values for *BRCA2* mutation carrier probability were: 1.0% for BOADICEA, 5.0% for BRCAPRO, and 5.5% for Penn II. Table S2 shows the numbers and proportions of *BRCA* mutation carriers in each predicted range category of the mutation carrier probability. BOADICEA gave good correlation between actual mutation rates and predicted mutation probability ranges. For the other models, the mutation rates had an upward trend with increasing ranges of predicted probabilities, but the actual rate values did not always fit the predicted probability ranges.

When the models were used to predict non-*BRCA* homologous recombination (HR) pathway gene mutation probabilities, the accuracies were generally poor (Fig. 1d). BRCAPRO was the only model that performed similarly to that of *BRCA2*, with AUC of 0.65 (95% CI, 0.56–0.73). The other models performed less well than that of *BRCA* genes, with AUC of 0.62 (95% CI, 0.53–0.71) for BOADICEA, 0.55 (95% CI, 0.46–0.65) for Myriad, and 0.54 (95% CI, 0.45–0.62) for Penn II.

### Model performance by clinical subgroups

**Figure 2:**
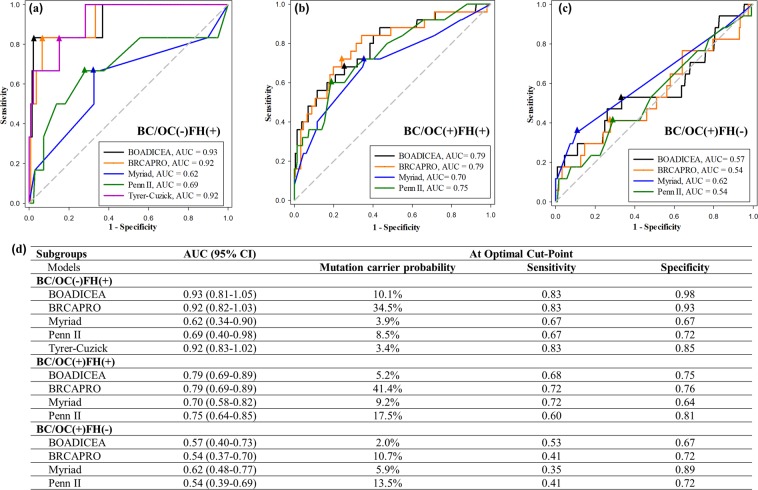
Performance of five *BRCA* mutation risk predictive models in the three subgroups using ROC curves; the respective area under the curve (AUC) for each model is shown at the right lower corner of the curves and in panel (**d**); the optimal cut-points (closest point to the left upper corner) are shown as triangles on each curve. (**a**) BC/OC(−)FH(+): in unaffected women with no personal history but with family history of breast or ovarian cancer, the probability of *BRCA1* or *BRCA2* mutation prediction using the BOADICEA, BRCAPRO, Myriad, Penn II and Tyrer-Cuzick models; (**b**) BC/OC(+)FH(+): in women with both personal history and family history of breast or ovarian cancer, the probability of *BRCA1* or *BRCA2* mutation prediction using the BOADICEA, BRCAPRO, Myriad, and Penn II models; (**c**) BC/OC(+)FH(−): in women with personal history but no family history of breast or ovarian cancer, the probability of *BRCA1* or *BRCA2* mutation prediction using the BOADICEA, BRCAPRO, Myriad, and Penn II models; (**d**) AUC with 95% confidence interval (CI) for each subgroup and each model, as well as mutation carrier probability, sensitivity, and specificity at the optimal cut-point for each curve are listed.

To find the population group where the models are the most applicable, we did subgroup analyses based on personal and familial BC/OC status. In the subgroup that had no personal history of BC/OC but had family history of BC/OC (designated BC/OC(−)FH(+) in Fig. 2a), three models had superb performance: AUC 0.93 (95% CI, 0.81–1.05) for BOADICEA, 0.92 (95% CI, 0.82–1.03) for BRCAPRO, and 0.92 (95% CI, 0.83–1.02) for the Tyrer-Cuzick model. In the BC/OC(+)FH(+) subgroup, the models performed fairly well with AUCs between 0.70 and 0.79 (Fig. 2b). In the BC/OC(+)FH(−) subgroup (Fig. 2c), the models had poor accuracy, except Myriad, which had comparable AUC (0.62) as those with family history (BC/OC(−)FH(+) 0.62 or BC/OC(+)FH(+) 0.70). The optimal cut-off values for mutation carrier probability were much higher for BRCAPRO than for all other models (Fig. 1d).

The observed and expected number of *BRCA* mutation carriers for the entire cohort and the subgroups are shown in Table 2. Examining the expected/observed (E/O) ratio, we found that the BOADICEA model gave a fairly accurate estimation of mutation rate (E/O 0.91) in the BC/OC(+)FH(+) subgroup but an underestimation in the other two subgroups. The Myriad prevalence table gave a good estimation of mutation rates in all subgroups (E/O 0.79 to 0.86), probably because the Myriad table was based on prevalence rates and was constructed by personal and family history of BC/OC, similar to our subgroup division. The BRCAPRO and Penn II models gave an approximately 2-fold overestimation in all subgroups.

**Table 2:** Comparison of observed and expected (model predicted) mutation proportions.

Subgroups	N	Obs	BOADICEA			BRCAPRO			Myriad			Penn II		
			AC	Exp	E/O	AC	Exp	E/O	AC	Exp	E/O	AC	Exp	E/O
All	647	48	5.0%	32.2	0.67	17.4%	112.5	2.34	6.1%	39.6	0.83	12.7%	82.3	1.71
BC/OC(+)FH(+)	241	25	9.4%	22.7	0.91	29.3%	70.6	2.83	8.8%	21.2	0.85	16.0%	38.6	1.54
BC/OC(+)FH(−)	262	17	2.3%	6.0	0.35	9.8%	25.7	1.51	5.1%	13.4	0.79	12.4%	32.5	1.91
BC/OC(−)FH(+)	144	6	2.5%	3.6	0.60	11.3%	16.3	2.71	3.6%	5.2	0.86	7.8%	11.2	1.87

Obs: observed number of mutation carriers; AC: predicted average carrier rate; Exp: expected number of mutation carriers = AC × N.

### Model performance by breast cancer pathology subtypes

**Figure 3:**
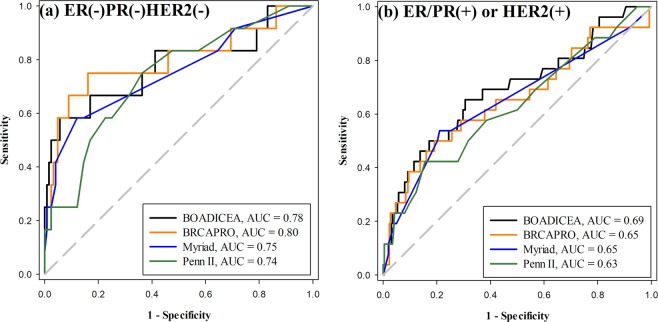
Performance of four *BRCA* mutation risk predictive models for women with breast cancer of different pathology subtypes (n = 424); the respective area under the ROC curve (AUC) for each model is shown at the right lower corner of the curves. (**a**) ER(−)PR(−)HER2(−) (triple negative) breast cancer, the probability of *BRCA1* or *BRCA2* mutation prediction using the BOADICEA, BRCAPRO, Myriad, and Penn II models; (**b**) ER/PR(+) (luminal type) or HER2(+) (HER2 overexpressed) breast cancer, the probability of *BRCA1* or *BRCA2* mutation prediction using the BOADICEA, BRCAPRO, Myriad, and Penn II models.

In our cohort, 424 women with personal history of breast cancer had estrogen receptor (ER), progesterone receptor (PR), and human epidermal growth factor receptor 2 (HER2) expression status of the breast tumor available. We divided these patients into three pathology subtypes based on the receptor status: triple negative (ER(−)PR(−)HER2(−)), luminal (ER or PR(+)HER2(−)), and HER2 overexpressed (HER2(+)) subtypes (Table 3). Figure 3 shows that the BOADICEA, BRCAPRO, Myriad, and Penn II models had better predictive accuracies in the triple negative breast cancer group (Fig. 3a, AUC 0.78, 0.80, 0.75, and 0.74, respectively) than in the luminal or HER2-overexpressed breast cancer group (Fig. 3b, AUC 0.69, 0.65, 0.65, and 0.63, respectively).

**Table 3:** Prediction accuracy of the BOADICEA model in women with breast cancer and available ER/PR/HER2 data, with and without incorporating receptor status.

Group	No.	*BRCA1/2* mutation rate	BOADICEA - AUC (95% CI)	
			w/o receptor status	w/ receptor status
All	424	38 (9.0%)	0.71 (0.61–0.81)	0.70 (0.59–0.80)
**By ER/PR/HER2 status**				
ER(−)PR(−)HER2(−)	136	12 (8.8%)	0.78 (0.60–0.95)	0.77 (0.58–0.95)
ER/PR(+)HER2(−)	209	22 (10.5%)	0.69 (0.56–0.82)	0.69 (0.55–0.82)
HER2(+)	79	4 (5.1%)	0.69 (0.36–1.00)	0.60 (0.23–0.97)
**By family history**				
BC(+)FH(+)	192	22 (11.5%)	0.78 (0.66–0.89)	0.76 (0.64–0.88)
BC(+)FH(−)	232	16 (6.9%)	0.60 (0.44–0.77)	0.59 (0.43–0.76)

**Figure 4:**
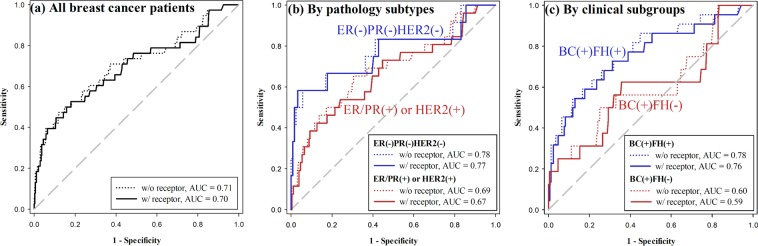
Performance of the BOADICEA model for women with breast cancer and ER/PR/HER2 status available (n = 424); the respective area under the ROC curve (AUC) for each subgroup is shown at the right lower corner of the curves. (**a**) For the entire group, the probability of *BRCA1* or *BRCA2* mutation prediction using BOADICEA without (dotted line) and with (solid line) incorporating the ER/PR/HER2 receptor status; (**b**) Comparison of pathology subtypes: triple negative ER(−)PR(−)HER2(−) (blue lines) and luminal ER/PR(+) or HER2(+) (red lines), the probability of *BRCA1* or *BRCA2* mutation prediction using BOADICEA without (dotted lines) and with (solid lines) incorporating the ER/PR/HER2 receptor status; (**c**) Comparison of clinical subgroups: BC(+)FH(+) (blue lines) and BC(+)FH(−) (red lines), the probability of *BRCA1* or *BRCA2* mutation prediction using BOADICEA without (dotted lines) and with (solid lines) incorporating the ER/PR/HER2 receptor status.

ER: estrogen receptor; PR: progesterone receptor; HER2: human epidermal growth factor receptor 2; ER/PR(+): ER(1+, 2+, or 3+) or PR(1+, 2+, or 3+); ER(−)PR(−): ER(−) and PR(−); HER2(+): HER2 overexpressed; HER2(−): HER2 not overexpressed. BC: breast cancer; BC(+): personal history of breast cancer; FH(+): family history of breast or ovarian cancer;

The BOADICEA model allows inclusion of the ER/PR/HER2 data in the calculation for *BRCA* mutation prediction. We compared the predictive accuracy with and without including the receptor status into the model, as shown in Table 3 and Fig. 4. The BOADICEA model performance did not improve with the additional receptor information for the group as a whole (AUC 0.71 to 0.70, Fig. 4a), or for any of the pathology or clinical subgroups (Fig. 4b,c).

### Setting the positive test threshold

The performance measures using sensitivity, specificity, positive predictive value (PPV) and negative predictive value (NPV) for each predictive model are shown in Table 4, with the positive test threshold for carrying a *BRCA1* or *BRCA2* mutation set at 10% or 20% carrier probability. For the entire study cohort, at the same threshold level, BRCAPRO had a relatively high sensitivity while BOADICEA had the highest specificity and PPV. Moving the threshold from 10% to 20%, the performance of BOADICEA and BRCAPRO were affected only slightly, while the performance measures of Penn II were significantly affected with a large drop in sensitivity (0.82 to 0.25) and a large rise in specificity (0.39 to 0.91). The positive predictive values (PPVs) were generally low due to the low mutation prevalence in the study cohort, although using the 20% threshold, BOADICEA could give a PPV of 0.46. The negative predictive values (NPVs) of all four models were high (range 0.94–0.96). In the BC/OC(-)FH(+) subgroup at a positive test threshold of 10%, Tyrer-Cuzick, BOADICEA, and BRCAPRO models gave good sensitivities (0.67, 0.83, and 0.83, respectively) and specificities (0.96, 0.98, and 0.70, respectively). Moving the threshold to 20% markedly lowered the sensitivity to 0.33 for Tyrer-Cuzick and BOADICEA without much gain in specificity. BRCAPRO’s performance did not differ much between the 10% and 20% thresholds.

**Table 4:** Performance measures for each model at the 10% or 20% threshold for *BRCA1/2* mutation carrier probability.

	Carrier number/rate	Test parameters at set threshold				
**Entire cohort**						
**10% threshold**	**<10%**	**≥10%**	**Sensitivity**	**Specificity**	**PPV**	**NPV**
BOADICEA	26/583	22/64	0.46	0.93	0.34	0.96
BRCAPRO	14/370	34/277	0.71	0.59	0.12	0.96
Myriad	25/526	23/121	0.48	0.84	0.19	0.95
Penn II	9/239	41/408	0.82	0.39	0.10	0.96
**20% threshold**	**<20%**	**≥20%**	**Sensitivity**	**Specificity**	**PPV**	**NPV**
BOADICEA	32/612	16/35	0.33	0.97	0.46	0.95
BRCAPRO	19/474	29/173	0.60	0.76	0.17	0.96
Myriad	37/612	11/35	0.23	0.96	0.31	0.94
Penn II	36/584	12/63	0.25	0.91	0.19	0.94
**BC/OC**(**−**)**FH**(+) **subgroup**						
**10% threshold**	**<10%**	**≥10%**	**Sensitivity**	**Specificity**	**PPV**	**NPV**
Tyrer-Cuzick	2/134	4/10	0.67	0.96	0.40	0.99
BOADICEA	1/136	5/8	0.83	0.98	0.62	0.99
BRCAPRO	1/97	5/47	0.83	0.70	0.11	0.99
Myriad	6/144	0/0	0.00	1.00	NaN	0.96
Penn II	3/28	3/116	0.50	0.18	0.03	0.89
**20% threshold**	**<20%**	**≥20%**	**Sensitivity**	**Specificity**	**PPV**	**NPV**
Tyrer-Cuzick	4/141	2/3	0.33	0.99	0.67	0.97
BOADICEA	4/141	2/3	0.33	0.99	0.67	0.97
BRCAPRO	1/112	5/32	0.83	0.80	0.16	0.99
Myriad	6/144	0/0	0.00	1.00	NaN	0.96
Penn II	6/142	0/2	0.00	0.99	0.00	0.96
**BC/OC**(+)**FH**(+) **subgroup**						
**10% threshold**	**<10%**	**≥10%**	**Sensitivity**	**Specificity**	**PPV**	**NPV**
BOADICEA	11/196	14/45	0.56	0.86	0.31	0.94
BRCAPRO	3/94	22/147	0.88	0.42	0.15	0.97
Myriad	7/145	18/96	0.72	0.64	0.19	0.95
Penn II	2/59	23/182	0.92	0.26	0.13	0.97
**20% threshold**	**<20%**	**≥20%**	**Sensitivity**	**Specificity**	**PPV**	**NPV**
BOADICEA	13/212	12/29	0.48	0.92	0.41	0.94
BRCAPRO	4/134	21/107	0.84	0.60	0.20	0.97
Myriad	16/208	9/33	0.36	0.89	0.27	0.92
Penn II	16/202	9/39	0.36	0.86	0.23	0.92
**BC/OC**(+)**FH**(**−**) **subgroup**						
**10% threshold**	**<10%**	**≥10%**	**Sensitivity**	**Specificity**	**PPV**	**NPV**
BOADICEA	14/253	3/9	0.18	0.98	0.33	0.94
BRCAPRO	10/180	7/82	0.41	0.69	0.09	0.94
Myriad	12/237	5/25	0.29	0.92	0.20	0.95
Penn II	4/64	13/198	0.76	0.24	0.07	0.94
**20% threshold**	**<20%**	**≥20%**	**Sensitivity**	**Specificity**	**PPV**	**NPV**
BOADICEA	15/259	2/3	0.12	1.00	0.67	0.94
BRCAPRO	14/228	3/34	0.18	0.87	0.09	0.94
Myriad	15/260	2/2	0.12	1.00	1.00	0.94
Penn II	14/240	3/22	0.18	0.92	0.14	0.94

PPV: positive predictive value; NPV: negative predictive value; NaN: not a number

## Discussion

Our study tested five widely-used *BRCA* mutation risk predictive models in a large Chinese cohort in Taiwan, which included breast or ovarian cancer patients with or without family history of breast or ovarian cancer, as well as unaffected women with family history of breast or ovarian cancer. Participants were enrolled to the study with comprehensive personal and pedigree data collection, and the same experimental and data analytical protocols for genetic testing were used for all participants. Our data thus allowed consistent evaluation of model performance not only in the cohort as a whole, but also in different subgroups of women with or without personal or family history of cancer. Using AUCs in the ROC curves for combined *BRCA1*/*2* predictions, we showed that BOADICEA and BRCAPRO models performed equally well in this Chinese cohort (AUCs 0.75 and 0.73) as previous studies in western cohorts (AUCs ranging from 0.71 to 0.77), while Myriad and Penn II models performed less well (AUCs 0.68 and 0.69) than those in western cohorts (AUCs ranging from 0.71–0.79)^16,27–30^.

In pre-test genetic counselling, it is important to know which model(s) are best used for which type of patients. We showed that BOADICEA and BRCAPRO were particularly well suited for unaffected women with family history (BC/OC(-)FH(+) subgroup), achieving AUCs of 0.93 and 0.92 respectively. These two models also performed fairly well (AUCs 0.79 and 0.79) for women with both personal and family history of breast or ovarian cancer (BC/OC(+)FH(+) subgroup), but they were close to unhelpful (AUCs 0.57 and 0.54) for those without family history. The Tyrer-Cuzick model can only be applied to unaffected women with family history and it worked very well for this subgroup and achieved AUC of 0.92. The Penn II and Myriad models worked the best for women with both personal and family history (AUC 0.75 and 0.70), and less well for unaffected women with family history (AUC 0.69 and 0.62). For women with breast or ovarian cancer but no family history, Myriad performed relatively well (AUC 0.62) compared to the other models.

Among women with breast cancer and known ER/PR/HER2 receptor status, we found that all models performed better for those with triple negative cancer than for those with luminal or HER2 overexpressed cancer, although none of the models actually used the receptor data in the prediction. In fact, incorporating the receptor status in the BOADICEA model had almost no effect on its predictive accuracy for the whole group or any of the subgroups.

All the models that predict separate *BRCA1* and *BRCA2* probabilities performed much better for *BRCA1* than for *BRCA2*: AUC of 0.98 vs 0.69 for BOADICEA, 0.93 vs 0.64 for BRCAPRO, and 0.85 vs 0.54 for Penn II. Similar difference was observed in previous studies but not as profound as our results^16,21,25,29^. This difference could possibly explain the poorer model performance in some Asian cohorts since *BRCA2* mutations seemed to be found more often than *BRCA1* mutations in Asians while it is the opposite in Whites^31–33^. Most models performed even poorer for non-*BRCA* HR pathway genes probably because these genes have lower penetrance in phenotype than *BRCA1/2*. However, BRCAPRO model gave similar AUC for these HR genes (0.65) to that of *BRCA2* (0.64).

A mutation risk threshold of 10% is often used to recommend genetic testing. The threshold could be set lower or higher depending on resources available to the individual or to the healthcare system. The availability of new cancer treatment options targeted for *BRCA*-mutated tumors, such as PARP inhibitors, could lower the threshold for testing. Moreover, the threshold should also depend on the model used to determine the risk. Our study showed that at the optimal points on the ROC curves, the cut-off carrier probability values were much higher for BRCAPRO (24.6%) than for BOADICEA (3.3%) (Fig. 1), consistent with the results shown in Table 2 that BRCAPRO seemed to overestimate mutation risk (E/O 2.34) while BOADICEA seemed to underestimate risk (E/O 0.67). Thus, setting a universal risk threshold for genetic testing may not be appropriate.

It is clear that most models rely heavily on family history of cancer. The models that performed better overall in the cohort (e.g. BOADICEA, BRCAPRO, Tyrer-Cuzick) utilized detailed pedigree data rather than the categorical yes/no or age cut-off clinical variables in the other models, while the models (e.g. Myriad) that incorporated more personal cancer information performed better for affected women without family history of breast or ovarian cancer. However, family histories are often limited due to many factors, including inaccurate or unavailable information, small families, scarcity of females in a family, premature death due to war or natural causes, migration or separation within a family. In modern societies, extended family history will probably become more and more limited. With more genetic testing results available, new models may be developed using personal history, and clinical and genetic information of nuclear families.

There are several limitations in our study. First, despite the relatively large Chinese cohort, our sample size was still limited. A larger pool of at risk individuals with genetic data available would make the model assessment more accurate, and new models could possibly be developed. Second, our cohort was consisted of a quite uniform Chinese population. The results may not be able to extend to other Asian ethnic groups. Third, the cohort included only women and a very small group of ovarian cancer patients, and therefore the results may not be applicable to men or women with ovarian cancer.

In summary, the five mutation predictive models performed generally well in this Chinese cohort as compared with western cohorts. The predictions were the most accurate for unaffected women with family history of breast or ovarian cancer using the BOADICEA, BRCAPRO and Tyrer-Cuzick models. The predictions were also fairly accurate for women with both personal and family history of breast or ovarian cancer, as well as for women with triple negative breast cancer. For breast or ovarian cancer patients with no family history, the predictions were quite unreliable. Between the two better-performed models, BOADICEA seemed to underestimate mutation risk while BRCAPRO seemed to overestimate mutation risk, thus we recommend setting higher risk threshold for genetic testing when using BRCAPRO (e.g. 20%) and lower risk threshold when using BOADICEA (e.g. 5%).

## Methods

### Study cohort and data collection

The study was conducted in accordance with the Declaration of Helsinki, and the study protocol was approved by the Institutional Review Board Committee at Koo Foundation Sun Yat-Sen Cancer Center (case No. 20141222A). Written informed consent was obtained from each study participant. Eligible individuals were enrolled between July 2015 and April 2017 at Koo Foundation Sun Yat-Sen Cancer Center (KF-SYSCC) to participate in germline testing of a panel of cancer susceptibility genes. Participants had to fulfil at least one of the following eligibility criteria: family history of breast or ovarian cancer at any age (2 or more individuals on the same lineage of the family), personal history of breast cancer or ovarian cancer with age of diagnosis less than or equal to 40, bilateral breast cancer, triple negative breast cancer, or both breast and ovarian cancer in the same individual. None of the participants had known mutation status in any cancer susceptibility genes prior to enrolment. Through participant surveys, detailed personal and family history regarding all cancers were collected, and pedigrees were extended to third-degree relatives as much as possible. The data of each pedigree was manually checked and formulated into a relational database for analysis. For the analyses in this study, male probands were excluded. ER, PR, and HER2 immunohistochemical (IHC) stains were available for a majority of invasive breast tumors in this cohort. ER(+) or PR(+) were defined as 1+, 2+ or 3+ on IHC stain. HER2(+) was defined as HER2 overexpression (3+ on IHC stain, or positive on dual *in situ* hybridization or fluorescence *in situ* hybridization).

### Genetic testing for *BRCA1/2* and other cancer susceptibility genes

Exonal and exon-flanking regions of twenty cancer susceptibility genes were sequenced on a next generation sequencing platform and variants were identified using standard protocols, details of which have been published previously^34^. We classified only protein-truncating variants including nonsense, frameshift, and splice-site mutations as pathogenic mutations. In addition to *BRCA1* and *BRCA2*, seven genes including *ATM*, *BARD1*, *BRIP1*, *PALB2*, *RAD50*, *RAD51C* and *RAD51D* were denoted as homologous recombination pathway genes for predictive model analyses.

### Calculation of germline mutation carrier probabilities

Relevant proband and pedigree information were formatted and stored in a local database, and input data for running the models were generated by in-house scripts (Perl, PHP, R script and shell script) in an automated fashion. Five mutation predictive models, BOADICEA, BRCAPRO, Myriad, Penn II, and Tyrer-Cuzick, were used for estimation of carrier probability of *BRCA* gene mutations. The prediction results were filtered and stored in the local database for statistical analyses.

The BOADICEA and BRCAPRO models compute the individual *BRCA* mutation carrier probability based on individual information on the proband and each of her relatives, including current age or age of death, incidence of breast, ovarian and other cancers, age at diagnosis and relationship to the proband. For BOADICEA, the predicted probability of carrying either a *BRCA1* or a *BRCA2* mutation was generated using the BOADICEA web application v3, (https://pluto.srl.cam.ac.uk/cgi-bin/bd3/v3/bd.cgi). BOADICEA allows input of the breast tumor ER/PR/HER2 (receptor) data. For comparison among the models, receptor status was not included in the calculation. Separate analyses comparing the predictive accuracies of BOADICEA with and without inclusion of the receptor status were performed. For BRCAPRO, the BayesMendel R package version 2.1–3 (available at https://projects.iq.harvard.edu/bayesmendel/bayesmendel-r-package) was used. The inputs for the Penn II and the Myriad model use a summary of personal and family cancer history. The Penn II predictions were carried out through a web interface (available at https://pennmodel2.pmacs.upenn.edu/penn2/). The Myriad model considers only the combined probability of carrying a mutation in either *BRCA1* or *BRCA2*, by using a mapping table downloaded from the Myriad Genetics website (https://myriadgenetics.eu/healthcare-professional-treating-diseases/hereditary-cancer-testing/hereditary-breast-and-ovarian-cancer-hboc-syndrom/prevalence-tables/). The Tyrer-Cuzick model is only applicable to individuals without personal history of breast or ovarian cancer but have family history of these cancers. The International Breast Cancer Intervention Study (IBIS) breast cancer risk evaluation tool (v8) was used to calculate the mutation carrier probabilities.

### Statistical analysis

Characteristics of the cohort were summarized using descriptive statistics stratified by subgroups. To determine the performance of the predictive models, receiver operating characteristics (ROC) curves were constructed, and the areas under the curve (AUCs) and 95% confidence interval (CI) were calculated. For each model, mutation carrier probability value, sensitivity, and specificity at the optimal cut-off point, which is the closest value to the left upper corner, were recorded. Subgroup analyses were done based on personal history and family history status. The applicable models were compared within the subgroups. Goodness of fit was assessed by comparing the expected/observed ratio (E/O) of the predicted probability to the actual frequency of the mutations, and calibration evaluated how well the model performed in each subgroup. Sensitivity, specificity, positive predictive value (PPV), and negative predictive value (NPV) were calculated for each risk model at the 10% and 20% thresholds for mutation carrier probability. All ROC curves were plotted using SigmaPlot (Systat Software, Inc.) version 12.0.

## Supplementary information

Supplementary Information
