# Supplementary material for: Evaluating BRCA mutation risk predictive models in a Chinese cohort in Taiwan
Source: Sci Rep. 2019 Jul 15;9:10229. doi: 10.1038/s41598-019-46707-6 (PMC6629692; doi:10.1038/s41598-019-46707-6)
Supplement: Supplementary file 1 — Supplementary Information [file 41598_2019_46707_MOESM1_ESM.pdf]

Evaluating *BRCA* mutation risk predictive models in a Chinese cohort in Taiwan

Fei-Hung Hung[2†], Yong Alison Wang[1,3*†], Jhih-Wei Jian[2], Hung-Pin Peng[2], Ling-Ling Hsieh[1], Chen-Fang Hung[1], Max M Yang[4], An-Suei Yang[2*]

[1]Koo Foundation Sun-Yat Sen Cancer Center, Taipei, Taiwan

[2]Genomic Research Center, Academia Sinica, Taipei, Taiwan

[3]National Yang Ming University School of Medicine, Taipei, Taiwan

[4]University of Cincinnati College of Medicine, Cincinnati, Ohio, U.S.A.

†Equal contribution

Supplementary Information

Table 1S. Number of individual gene mutation carriers in the entire cohort and the three clinical subgroups

Table 2S. *BRCA* mutation carrier rates according to mutation carrier probability categories predicted by the models

Table 1S. Number of individual gene mutation carriers in the entire cohort and the three clinical subgroups

Mutations detected, no (%)	All Participants N=647	BC/OC(+)FH(+) n=241	BC/OC(+)FH(-) n=262	BC/OC(-)FH(+) n=144
BRCA1 mutation	12 (1.9%)	8 (3.3%)	2 (0.8%)	2 (1.4%)
BRCA2 mutation	36 (5.6%)	17 (7.1%)	15 (5.7%)	4 (2.8%)
Other HR gene mutations	31 (4.8%)	14 (5.8%)	10 (3.8%)	7 (4.9%)
ATM	9 (1.2%)	4 (1.7%)	1 (0.4%)	4 (2.8%)
BARD1	1 (0.2%)	1 (0.4%)	0 (0%)	0 (0%)
BRIP1	1 (0.2%)	1 (0.4%)	0 (0%)	0 (0%)
PALB2	10 (1.5%)	5 (2.1%)	3 (1.1%)	2 (1.8%)
RAD50	1 (0.2%)	1 (0.4%)	0 (0%)	0 (0%)
RAD51C	3 (0.5%)	0 (0%)	2 (0.8%)	1 (0.7%)
RAD51D	6 (0.9%)	2 (0.8%)	4 (1.5%)	0 (%)

Table 2S. *BRCA* mutation carrier rates according to mutation carrier probability categories predicted by the models

	Category of predicted *BRCA1/2* mutation carrier probability (%)					
	<10%	10-20%	20-40%	40-60%	60-80%	>80%
**BOADICEA**						
BRCA1/2	26/583 (4.5%)	6/29 (20.7%)	6/19 (31.6%)	4/7 (57.1%)	1/3 (33.3%)	5/6 (83.3%)
BRCA1	2/607 (0.3%)	0/15 (0.0%)	5/16 (31.3%)	0/2 (0.0%)	1/3(33.3%)	4/4 (100.0%)
BRCA2	33/628 (5.3%)	1/13 (7.7%)	0/3 (0.0%)	2/3 (66.7%)	0/0 (N/A)	0/0 (N/A)
**BRCAPRO**						
BRCA1/2	14/370 (3.8%)	5/104 (4.8%)	5/81 (6.2%)	9/37 (24.3%)	5/32 (15.6%)	10/23 (43.5%)
BRCA1	1/477 (0.2%)	1/84 (1.2%)	4/51 (7.8%)	0/19 (0.0%)	2/10 (20.0%)	4/6 (66.7%)
BRCA2	19/494 (3.8%)	7/77 (9.1%)	8/62 (12.9%)	1/12 (8.3%)	1/2 (50%)	0/0 (N/A)
**Myriad**						
BRCA1/2	25/526 (4.8%)	12/86 (14.0%)	9/33 (27.3%)	2/2 (100.0%)	0/0 (N/A)	0/0 (N/A)
**Penn II**						
BRCA1/2	9/239 (3.8%)	27/345 (7.8%)	6/53 (11.3%)	2/4 (50.0%)	0/2 (0.0%)	4/4 (100.0%)
BRCA1	4/553 (0.7%)	2/75 (2.7%)	2/11 (18.2%)	0/4 (0.0%)	0/0 (N/A)	4/4 (100.0%)
BRCA2	31/611 (5.1%)	4/32 (12.5%)	1/4 (25.0%)	0/0 (N/A)	0/0 (N/A)	0/0 (N/A)

N/A: not applicable
